# HIV and HCV prevalence among entrants to methadone maintenance treatment clinics in China: a systematic review and meta-analysis

**DOI:** 10.1186/1471-2334-12-130

**Published:** 2012-06-08

**Authors:** Xun Zhuang, Yanxian Liang, Eric PF Chow, Yafei Wang, David P Wilson, Lei Zhang

**Affiliations:** 1 School of Public Health, Nantong University, Jiangsu Province, China; 2 The Kirby Institute, University of New South Wales, Sydney, Australia

**Keywords:** HIV, HCV, Co-infection, Prevalence, Methadone maintenance treatment, Meta-analysis, China

## Abstract

**Background:**

Methadone maintenance treatment (MMT) was implemented in China since 2004. It was initiated in 8 pilot clinics and subsequently expanded to 738 clinics by the end of 2011. Numerous individual research studies have been conducted to estimate HIV and HCV prevalence among MMT clients but an overview of the epidemics in relations to MMT remains unclear. The aim of this study is to estimate the magnitude and changing trends of HIV, HCV and HIV-HCV co-infections among entry clients to MMT clinics in China during 2004-2010.

**Methods:**

Chinese and English databases of literature were searched for studies reporting HIV, HCV and co-infection prevalence among MMT clients in China from 2004 to 2010. The prevalence estimates were summarized through a systematic review and meta-analysis of published literatures.

**Results:**

Ninety eligible articles were selected in this review (2 in English and 88 in Chinese). Nationally, pooled prevalence of HIV-HCV and HIV-HCV co-infection among MMT clients was 6.0% (95%CI: 4.7%-7.7%), 60.1% (95%CI: 52.8%-67.0%) and 4.6% (95%CI: 2.9%-7.2%), respectively. No significant temporal trend was found in pooled prevalence estimates. Study location is the major contributor of heterogeneities of both HIV and HCV prevalence among drug users in MMT.

**Conclusions:**

There was no significant temporal trend in HIV and HCV prevalence among clients in MMT during 2004–2010. Prevalence of HCV is markedly higher than prevalence of HIV among MMT clients. It is recommended that health educational programs in China promote the earlier initiation and wider coverage of MMT among injecting drug users (IDUs), especially HIV-infected IDUs.

## Background

Since the implementation of open-door policies in 1979, illicit drug trade has re-emerged in China. As the most populous country in the world, China has observed a rapid increase in drug use over the past three decades [1]. The number of officially registered drug users increased from 70,000 in 1990 to 1.33 million at the end of 2009 [2]. Moreover, behind each registered drug user, there were estimated 2–4 implicit drug users [3]. Official statistics indicates that 73.2% of drug users in China in 2009 used heroin [2]. Intravenous injection is the most common mean of drug use, with injecting drug users (IDUs) accounting for 59–85% of drug users [4-10].

Drug users (DUs), especially IDUs, represent a high-risk population for spreading HIV infection due to their high frequency of injection, sharing of contaminated needles and other risk behaviours [11-15]. The first domestic Chinese HIV/AIDS cases were found among heroin users in Yunnan Province as early as 1989 [16]. By the end of 2002, HIV cases among drug users were reported in all 31 Chinese provinces, autonomous regions and municipalities [4]. The cumulative number of diagnosed HIV/AIDS cases in China is now well over 200,000, among which over 60% were drug users [6,17]. IDUs account for over 40% of new HIV infections [6,17].

In China, HIV prevalence has wide geographic variations. According to the Chinese national surveillance report for 1995–2009 [18], five provinces (Yunnan, Guizhou, Sichuan, Guangxi and Xinjiang) were classified as high transmission areas (HTAs) for HIV infection among drug users, and the rest of country was considered as low transmission areas (LTAs). HIV prevalence among drug users in Yunnan and Xinjiang were the highest among all Chinese provinces, of approximately 25%-30% in 2007 [18,19]. Similarly, HCV prevalence among DUs in China is high, but estimates vary substantially from 15.6% to 98.7% [20-23].

Harm reduction programs for drug users began in 2003 in China and it is believed that they have contributed to reductions in the spread of HIV [24-26]. A major component of this program is methadone maintenance treatment (MMT), which was initiated as a pilot program in 8 clinics serving 1,029 drug users in 2004 and subsequently expanded to 738 clinics serving 344,254 drug users by the end of 2011, which accounts for approximately 30% of registered IDUs in China (personal communication with China CDC). Initially, strict enrolment criteria were imposed to enroll only registered drug users [27]. In July 2006, new implementation protocol of the community-based MMT program has been announced by the Chinese Ministry of Health to cover a wider group of drug users [28]. The new protocol recommended MMT clinics also offer ancillary services including counseling and psychosocial support, testing for HIV and infections, referrals for antiretroviral treatment and other social supports [27].

There have been numerous independent studies documenting the prevalence of HIV or HCV among MMT clients at baseline of their treatment. Across these studies, large variations in HIV and HCV prevalence estimates were commonly observed, reflecting the complex geographical and chronological overlapping of the two epidemics. Numerous individual research studies have been conducted to estimate HIV and HCV prevalence among MMT clients in China. The studies vary in time and geographical locations, and do not provide a complete overview of HIV and HCV epidemics in relations to MMT in China. This study aims to investigate the geographical and the temporal patterns of HIV and HCV epidemics in China and their likely interaction. We do this through a systematic review and meta-analysis and we also discuss implications for future MMT implementation and health policies for HIV prevention among IDUs in China.

## Methods

### Search strategy

Two independent investigators conducted a systematic review of published peer-reviewed research articles by searching the following databases: PubMed, Chinese Scientific Journals Fulltext Database (CQVIP), China National Knowledge Infrastructure (CNKI) and Wanfang Data from 2004–2010. Keywords used in the database search included (“Methadone” *OR* “Methadone Maintenance Treatment” *OR* “Methadone Maintenance Therapy” *OR* “Methadone Maintenance”) *AND* (“HIV” *OR* “AIDS” *OR* “HCV” *OR* “hepatitis C virus” *OR* “co-infection”) *AND* (“China” *OR* “China Mainland”). We also performed a manual search of the reference lists of published articles. This review was conducted and reported according to the PRISMA (Preferred Reporting Items for Systematic Reviews and Meta-Analyses) Statement issued in 2009 [29].

### Study selection

Studies were eligible for inclusion in this systematic review if they met the following criteria: (1) study published in Chinese or English language; (2) study reported HIV or HCV prevalence estimates among clients in MMT at baseline of treatment in China; (3) HIV and HCV infection must be diagnosed from laboratory serologic testing; (4) study design such as study site, time period and sample size must be reported. Intervention studies among MMT clients were also included. Exclusion criteria were: (1) review papers; (2) non peer–reviewed local/government reports; (3) conference abstracts and presentations; (4) self-reported HIV or HCV infections; (5) dissertations. If the same study data were published in both English and Chinese sources, the articles published in Chinese language were excluded from this study.

### Validity assessment

The quality of studies was assessed using a validated quality assessment tool for cross-sectional studies [30]. The following eight items were assessed to calculate a total quality score: (1) clear definition of the target population; (2) representativeness of probability sampling; (3) sample characteristics matching the overall population; (4) adequate response rate; (5) standardized data collection methods; (6) reliable of survey measures/instruments; (7) valid of survey measures/instruments; (8) appropriate statistical methods. Answers were scored 0 and 1 for ‘No’ and ‘Yes’, respectively. The total quality score varied between 0 and 8 for each study.

### Data abstraction

We extracted the following information from all eligible studies: published year, study site, study period, gender, age, marital status, education, study design, clients recruitment methods, sample size; laboratory test methods for HIV and HCV, prevalence of HIV, HCV and HIV-HCV co-infection among MMT clients at the baseline of the treatment. The studies were then categorized by geographical locations according to their level of HIV transmission, and also into two specific time periods, prior to and after 2006.

### Statistical analysis

Meta-analyses were carried out with the Comprehensive Meta-Analysis software (V2.0, Biostat, Englewood, New Jersey). The effect rates of pooled prevalence estimates and 95% confidence intervals (CI) were determined based on random effect models. Random effect models were applied when heterogeneity across subgroups were found to be significant. Heterogeneity tests were performed using the Cochran Q-test (*p* < 0.10 represents statistically significant heterogeneity) and *I*^2^ statistic. We investigated the factors that are associated with heterogeneities in the stratified meta-analyses using meta-regression. Meta-regression was performed in STATA 10.0 (StataCrop, Texas, USA) Potential publication bias was measured by the Begg and Mazumdar rank correlation (*p* < 0.05). Spearmen correlation was used to assess the relationship between HIV prevalence and HCV prevalence among clients in MMT in China.

## Results

### Trial flow/flow of included studies

**Figure 1:**
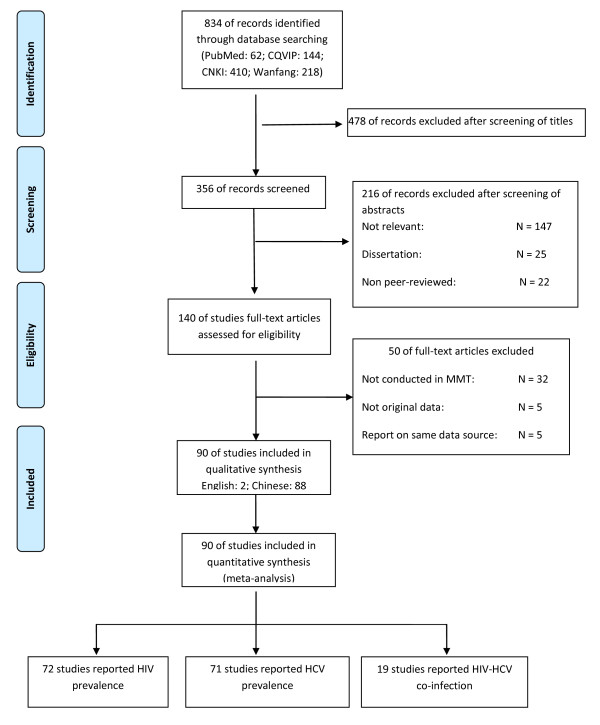
**Flow chart showing the meta-analysis studies selection.** N; the number of articles included in systematic review; n, the number of prevalence estimates included in meta-analysis.

A total of 834 studies were identified from four electronic databases (62 in PubMed, 410 in CNKI, 144 in CQVIP, 218 in Wanfang database). Due to duplication and irrelevance, we excluded 478 articles after screening the titles. We screened the abstracts of the remaining 356 articles, following which 216 articles were excluded (147 were not relevant, 25 were dissertations, 22 were non peer-reviewed theses, 10 were conference presentations or abstracts, 7 were reviews or letters and 5 were not carried out in mainland China). Among the remaining 140 articles eligible for full-text screening; we further excluded 50 articles (32 were not conducted among MMT clients, 5 did not report original data, 5 reported results from already identified data sources, 3 did not report study sites, 3 did not report study period, 2 studies covered multiple provinces). We finally included 90 articles (2 in English and 88 in Chinese) for subsequent quantitative synthesis, of which, 72, 71 and 19 articles were eligible for meta-analysis of HIV, HCV and HIV-HCV co-infection prevalence, respectively. The selection process is illustrated in Figure 1 and the quality score of each study is shown in the Additional file 1: Table S1.

### Study characteristics

The 90 eligible articles were from 19 of the 31 Chinese provinces. The sample size of the selected studies ranged from 38 to 8662 (median: 300; IQR: 175–512). All participants were recruited from MMT clinics. Eighty-one out of 90 studies (90%) were cross-sectional, 5 (6%) were cohort studies, 2 (2%) were randomized controlled trials and 2 (2%) did not report the study design. There were 72, 71 and 19 prevalence estimates on HIV, HCV and HIV-HCV co-infection among MMT clients at baseline of treatment respectively.

### Epidemic trends of HIV, HCV infection and HIV-HCV co-infection

**Figure 2:**
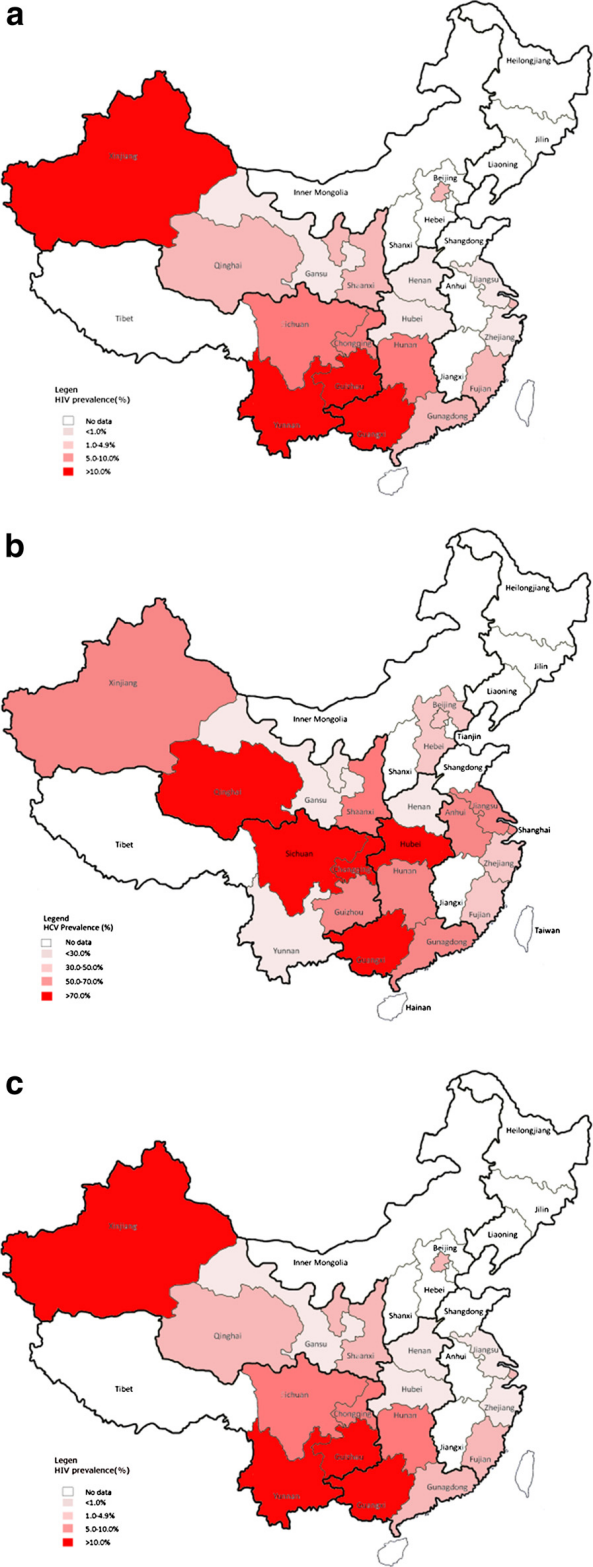
The regional distribution of pooled prevalence of (a) HIV infection; (b) HCV infection; and (c) HIV-HCV co-infection in MMT in China.

The overall national prevalence of HIV, HCV and HIV-HCV co-infection among MMT clients at baseline of treatment in China were 6.0% (95% CI: 4.7-7.7%), 60.1% (95% CI: 52.8-67.0%) and 4.6% (95% CI: 2.9-7.2%) respectively. Prevalence in HTAs were consistently higher than those in LTAs (HIV: 17.5% (95% CI: 14.0-21.6%) vs. 2.4% (95% CI: 1.6-3.5%); HCV: 70.2% (95% CI: 62.6-76.8%) vs. 55.5% (95% CI: 46.4-64.2%); HIV-HCV co-infection: 8.0% (95% CI: 4.8-12.9%) vs. 2.5% (95% CI: 1.4-4.4%)). Large variations in prevalence were also observed geographically. Whereas HIV prevalence among MMT clients varied from 0.1% (Hubei) to 36.0% (Yunnan) (Table 1, Figure 2a), HCV prevalence varied from 10.9% (Henan) to 65.7% (Guangxi) (Table 2, Figure 2b) and HIV-HCV co-infection varied from 0.5% (Henan) to 12.6% (Guangxi) (Table 3, Figure 2c). The forest plots showing the results of meta-analysis of HIV, HCV and HIV-HCV co-infection prevalence among clients in MMT are illustrated in the Additional file 1: Figure S1, S2 and S3. However, no temporal trends of any of these infections were found at the national level, in HTAs or LTAs since the initiation of MMT in 2004.

**Table 1:** Summary of HIV prevalence among clients in MMT in China

**Groups**	**Provinces**	**No of studies**	**Study Year**	**Prevalence (individual studies) (95% CI)**	**Pooled prevalence (provinces) (95% CI)**	**Pooled prevalence (group) (95% CI)**
HTAs	Guangxi	3			17.7%(7.2%-37.4%)	17.5% (14.0%-21.6%)
	(ZhouXP,2009[31]		2007	28.2%(22.0%-35.5%)		
	ChangZR,2010[32]		2008	27.9%(25.2%-30.7%)		
	BaiY,2009[33])		2009	6.0%(4.2%-8.4%)		
	Guizhou	4			20.7%(14.2%-29.2%)	
	(ChangZR,2010[32]		2008	24.6%(21.5%-28.1%)		
	HanXJ,2010[34]		2009	27.0%(22.3%-32.2%)		
	LiXY,2009[35]		2009	0.5%(0.1%-3.5%)		
	WangJ,2010[36])		2009	20.3%(14.5%-27.7%)		
	Sichuan	8			7.1%(3.8%-12.9%)	
	(HaoC,2006[37]		2004	13.6%(10.4%-17.6%)		
	QianHZ,2007[38]		2005	14.0%(11.4%-17.1%)		
	ChenB,2009[39]		2006	2.0%(1.0%-3.9%)		
	LiuJK,2009[40]		2006	14.1%(8.2%-23.2%)		
	DongG,2009[41]		2007	4.3.%(2.6%-7.0%)		
	WangY,2009[42]		2008	3.8%(2.6%-5.5%)		
	WangDY,2009[43]		2008	21.7%(18.9%-24.9%)		
	YaoW,2008[44])		2008	2.3%(1.4%-3.6%)		
	Xinjiang	6			22.0%(15.2%-30.6%)	
	(LiuJB,2006[45]		2005	19.4%(13.4%-27.1%)		
	FuLP,2007[46]		2006	33.4%(29.7%-37.3%)		
	FangHR,2008[47]		2008	14.2%(12.6%-16.1%)		
	ReZW,2009[48]		2008	28.6%(22.4.5%-35.7%)		
	ShenL,2009[49]		2008	14.9%(12.8%-17.3%)		
	YuanL,2010[50])		2008	26.55(20.8%-33.0%)		
	Yunnan	6			36.0%(25.6%-47.9%)	
	(DuanYJ,2008[51]		2006	33.3%(24.8%-43.2%)		
	ZhangMJ,2008[52]		2007	51.8%(44.2%-59.3%)		
	ZhuangHY,2008[53]		2007	66.7%(51.8%-78.8%)		
	XueHM,2010[54]		2008	39.3%(37.1%-41.5%)		
	YangGW,2010[55]		2008	15.7%(11.4%-21.3%)		
	YangYC,2011[56])		2009	23.1%(21.7%-24.5%)		
LTAs	Beijing	1			4.6%(2.7%-7.8%)	2.4%(1.6%-3.5%)
	(DuWJ,2007[57])		2005	4.6%(2.7%-7.8%)		
	Chongqing	3			6.8%(4.3%-10.6%)	
	(TanXL,2007[58]		2005	7.8%(5.5%-11.0%)		
	WuGH,2010[59]		2007	9.2%(7.1%-11.8%)		
	ZhouX,2009[60])		2008	4.3%(3.0%-6.1%)		
	Fujian	2			2.1%(1.1%-3.9%)	
	(WuLH,2007[61]		2006	2.0%(0.5%-7.6%)		
	ZhengWX,2009[62])		2006	2.1%(1.0%-4.3%)		
	Gansu	2			0.7%(0.3%-1.5%)	
	(GaoLF,2010[63]		2009	0.8%(0.2%-3.2%)		
	ZhuXH,2010[64])		2010	0.7%(0.2%-1.7%)		
	Guangdong	13			3.6%(1.6%-8.3%)	
	(DaiLP,2009[65]		2007	5.9%(2.7%-12.6%)		
	LiuXY,2009[66]		2007	1.2%(1.0%-1.5%)		
	ZhangQL,2008[67]		2007	0.2%(0.0%-3.8%)		
	ChenW,2009[68]		2008	20.7%(17.2%-24.7%)		
	ChenA,2007[69]		2008	19.6%(16.4%-23.3%)		
	DaiLP,2010[70]		2008	3.5%(1.8%-6.6%)		
	HuWS,2010[71]		2008	9.6%(5.5%-16.1%)		
	LiLY,2009[72]		2008	1.0%(0.1%-6.85)		
	LiYF,2009[73]		2008	2.5%(1.0%-5.9%)		
	WangM,2009[74]		2008	1.3%(0.1%-17.5%)		
	WangCQ,2009[75]		2009	1.9%(1.0%-3.8%)		
	WuZL,2010[76]		2009	7.9%(5.8%-10.6%)		
	XiaL,2010[77])		2009	1.2%(0.05%-3.8%)		
	Henan	1			0.9%(0.2%-3.6%)	
	(WuSX,2009[78])		2008	0.9%(0.2%-3.6%)		
	Hubei	1			0.1%(0.0%-1.8%)	
	(QiuXQ,2009[79])		2006	0.1%(0.0%-1.8%)		
	Hunan	7			6.9%(4.1%-11.4%)	
	(LiXL,2008[80]		2006	1.2%(0.3%-4.7%)		
	LiXL,2009[81]		2006	16.7%(14.2%-19.7%)		
	TangXY,2007[82]		2006	14.7%(10.2%-20.7%)		
	ChenLF,2009[83]		2007	13.7%(10.3%-18.1%)		
	HeHX,2008[84]		2007	9.5%(7.5%-12.0%)		
	ChenC,2010[85]		2008	1.9%(0.5%-7.2%)		
	FengYH,2010[86])		2009	0.6%(0.2%-1.8%)		
	Jiangsu	6			0.9%(0.5%-1.5%)	
	(WangYP,2009[87]		2007	1.4%(0.5%-4.3%)		
	FengSQ,2010[88]		2008	1.1%(0.4%-2.8%)		
	HaoC,2009[89]		2008	0.6%(0.2%-1.6%)		
	XuGY,2008[90]		2008	0.5%(0.0%-7.3%)		
	ZhangMH,2010[91]		2008	1.0%(0.3%-3.0%)		
	YuanZX,2010[92])		2009	0.2%(0.0%-3.4%)		
	Ningxia	1			1.2%(0.5%-2.6%)	
	(JiangA,2009[93])		2007	1.2%(0.5%-2.6%)		
	Qinghai	1			1.4%(0.5%-3.8%)	
	(HaoXQ,2009[94])		2008	1.4%(0.5%-3.8%)		
	Shaanxi	2			1.4%(0.5%-3.8%)	
	(JiaW,2008[95]		2006	1.7%(0.75%-3.9%)		
	ZhangHF,2009[96])		2008	0.4%(0.0%-6.3%)		
	Shanghai	2			1.4%(0.5%-4.8%)	
	(LiuY,2009[97]		2008	1.5%(0.5%-4.4%)		
	LiT,2010[98])		2009	1.0%(0.1%-13.6%)		
	Zhejiang	3			0.5%(0.1%-1.6%)	
	(CaiCP,2008[99]		2007	0.6%(0.0%-8.4%)		
	ZhangXH,2008[100]		2007	0.6%(0.1%-4.2%)		
	SuMF,2010[101])		2009	0.3%(0.0%-2.3%)		

**Table 2:** Summary of HCV prevalence among clients in MMT in China

**Groups**	**Provinces**	**No of studies**	**Study Year**	**Prevalence (individual studies) (95% CI)**	**Pooled prevalence (provinces) (95% CI)**	**Pooled prevalence (group) (95% CI)**
HTAs	Guangxi	3			87.2%(85.4%-88.8%)	70.2%(62.6%-76.8%)
	(ZhouXP,2009[31]		2007	88.8%(83.1%-92.8%)		
	ChangZR,2010[32]		2008	87.9%(85.5%-89.8%)		
	BaiY,2009[33])		2009	85.5%(82.2%-88.3%)		
	Guizhou	6			61.5%(46.1%-75.0%)	
	(ChangZR,2010[32]		2008	76.1%(72.7%-79.3%)		
	WangJ,2008[102]		2008	41.6%(34.6%-48.9%)		
	DengCK,2009[103]		2008	61.4%(55.9%-66.7%)		
	HanXJ,2010[34]		2009	60.0%(54.4%-65.4%)		
	LiXY,2009[35]		2009	33.5%(27.3%-40.4%)		
	WangJ,2010[36])		2009	86.7%(80.1%-91.4%)		
	Sichuan	6			77.4%(71.0%-82.8%)	
	(HaoC, 2006[37]		2004	67.3%(62.2%-72.1%)		
	QianHZ,2007[38]		2005	68.4%(64.4%-72.1%)		
	DongG,2009[41]		2007	78.6%(74.0%-82.6%)		
	WangDY,2009[43]		2008	83.7%(80.7%-86.2%)		
	YaoW,2008[44]		2008	82.9%(80.0%-85.4%)		
	ZhaoXH,2009[104])		2008	80.1%(75.9%-83.7%)		
	Xinjiang	5			64.8%(50.0%-77.2%)	
	(LiuJB,2006[45]		2005	69.0%(60.5%-76.4%)		
	FuLP,2007[46]		2006	40.0%(36.1%-44.0%)		
	FangHR,2008[47]		2008	68.9%(66.5%-71.2%)		
	YuanL,2010[50]		2008	72.0%(65.4%-77.8%)		
	ReZW,2009[48])		2008	72.0%(64.9%-78.2%)		
	Yunnan	1			23.8%(18.5%-30.0%)	
	(YangGW,2010[55])		2008	23.8%(18.5%-30.0%)		
LTAs	Anhui	2			62.3%(17.0%-93.0%)	55.5%(46.4%-64.2%)
	(WangXR,2007[105]		2007	36.4%(31.0%-42.2%)		
	ZhanSW,2008[106])		2007	82.8%(77.1%-87.3%)		
	Beijing	1			46.4%(40.7%-52.3%)	
	(DuWJ,2007[57])		2005	46.4%(40.7%-52.3%)		
	Chongqing	2			76.2%(73.6%-78.5%)	
	(ZhouX,2009[60]		2007	74.7%(71.0%-78.1%)		
	WuGH,2010[59])		2008	77.2%(74.3%-79.9%)		
	Fujian	2			48.4%(26.2%-71.1%)	
	(WuLH,2007[61]		2006	36.0%(27.2%-45.8%)		
	ZhengWX,2009[62])		2006	60.2%(54.8%-65.3%)		
	Gansu	3			23.7%(18.0%-30.7%)	
	(GaoLF,2010[63]		2009	23.8%(18.9%-29.5%)		
	HeXX,2010[107]		2009	19.5%(17.6%-21.6%)		
	ZhuXH,2010[64])		2010	28.7%(25.3%-32.4%)		
	Guangdog	8			63.7%(25.2%-90.1%)	
	(LiuXY,2009[66]		2007	5.4%(4.9%-5.9%)		
	ZhangQL,2008[67]		2007	30.3%(24.4%-37.0%)		
	HuWS,2010[71]		2008	96.0%(90.7%-98.3%)		
	LiLY,2009[72]		2008	67.0%(57.2%-75.5%)		
	LiYF,2009[73]		2008	77.0%(70.7%-82.3%)		
	WangM,2009[74]		2008	81.6%(66.1%-91.0%)		
	WangCQ,2009[75]		2009	77.3%(73.0%-81.0%)		
	XiaL,2010[77])		2009	62.2%(56.1%-68.1%)		
	Henan	1			10.9%(7.4%-15.8%)	
	(WuSX,2009[78])		2008	10.9%(7.4%-15.8%)		
	Hubei	1			94.3%(91.2%-96.3%)	
	(PengJS,2007[108])		2006	94.3%(91.2%-96.3%)		
	Hunan	5			66.4%(49.3%-80.1%)	
	(LiXL,2008[80]		2006	49.4%(41.8%-57.0%)		
	HeHX,2008[84]		2007	58.3%(54.5%-62.0%)		
	ChenLF,2009[83]		2007	59.5%(54.9%-63.9%)		
	ChenC,2010[85]		2008	51.4%(42.0%-60.7%)		
	FengYH,2010[86])		2009	93.7%(91.2%-95.5%)		
	Jiangsu	10			61.6%(51.8%-70.6%)	
	(XiaX,2007[109]		2006	35.0%(32.6%-37.6%)		
	XuYP,2007[110]		2006	61.0%(56.1%-65.6%)		
	WangYP,2009[87]		2007	70.5%(64.0%-76.3%)		
	FengSQ,2010[88]		2008	52.3%(47.2%-57.3%)		
	HaoC,2009[89]		2008	51.8%(47.9%-55.6%)		
	SongHB,2010[111]		2008	66.2%(59.6%-72.2%)		
	XuGY,2008[90]		2008	76.2%(67.0%-83.5%)		
	ZhangMH,2010[91]		2008	75.1%(70.0%-79.6%)		
	YuanZX,2010[92]		2009	56.5%(50.1%-63.0%)		
	WangWM,2010[112])		2010	68.8%(62.3%-74.7%)		
	Ningxia	2			23.0%(16.7%-30.9%)	
	(JiangA,2009[93]		2007	20.3%(17.0%-24.0%)		
	LiuXP,2010[113])		2009	28.0%(19.8%-37.9%)		
	Qinghai	1			70.8%(65.1%-75.8%)	
	(HaoXQ,2009[94])		2008	70.8%(65.1%-75.8%)		
	Shaanxi				55.3%(43.2%-66.7%)	
	(JiaW,2008[95]	5	2006	54.0%(48.3%-59.65)		
	LiYC,2009[114]		2008	60.6%(55.8%-65.3%)		
	ZhangHF,2009[96]		2008	40.0%(31.6%-49.0%)		
	ZangJF,2010[115]		2009	42.6%(30.9%-55.2%)		
	LiuHB,2010[116])		2010	73.8%(69.7%-77.5%)		
	Shanghai	2			59.7%(54.2%-64.9%)	
	(LiuY,2009[97]		2008	61.2%(54.3%-67.6%)		
	DuJ,2009[117])		2009	57.0%(47.8%-65.8%)		
	Zhejiang	5			40.1%(24.75%-57.8%)	
	(ZhangXH,2007[118]		2006	28.9%(16.8%-45.1%)		
	CaiCP,2008[99]		2007	75.9%(65.8%-83.7%)		
	ZhangXH,2008[100]		2007	40.6%(33.4%-48.3%)		
	FuYF,2009[119]		2008	40.4%(35.4%-45.65%)		
	SuMF,2010[101])		2009	18.9%(14.8%-23.7%)		

**Table 3:** Summary of HIV-HCV Co-infection among clients in MMT in China

**Groups**	**Provinces**	**No of studies**	**Study Year**	**Prevalence (individual studies) (95% CI)**	**Pooled prevalence (provinces) (95%CI)**	**Pooled prevalence (group) (95% CI)**
HTAs	Guangxi	3			12.6%(4.0%-33.4%)	8.0%(4.8%-12.9%)
	(ZhouXP,2009[31]		2007	11.2%(7.2%-16.9%)		
	ChangZR,2010[32]		2008	27.4%(24.7%-30.2%)		
	BaiY,2009[33])		2009	5.8%(4.1%-8.2%)		
	Guizhou	3			3.8%(0.4%-29.3%)	
	(ChangZR,2010[32]		2008	23.1%(20.0%-26.5%)		
	WangJ,2010[36]		2009	0.7%(0.1%-4.8%)		
	HanXJ,2010[34])		2009	2.0%(0.9%-4.4%)		
	Sichuan	4			6.8%(2.8%-15.8%)	
	(QianHZ,2007[38]		2005	13.5%(10.9%-16.6%)		
	DongG,2009[41]		2007	3.4%(2.0%-5.9%)		
	WangDY,2010[120]		2008	19.2%(16.4%-22.35%)		
	YaoW,2008[44])		2008	1.9%(1.1%-3.2%)		
LTAs	Chongqing	2			5.3%(1.9%-13.9%)	2.5%(1.4%-4.4%)
	(ZhouX,2009[60]		2007	8.7%(6.6%-11.2%)		
	WuGH,2010[59])		2008	3.1%(2.1%-4.5%)		
	Fujian	1			1.0%(0.1%-6.8%)	
	(WuLH,2007[61])		2006	1.0%(0.1%-6.8%)		
	Gansu	1			0.8%(0.2%-3.2%)	
	(GaoLF,2010[63])		2009	0.8%(0.2%-3.2%)		
	Guangdong	2			1.9%(0.9%-3.8%)	
	(LIYF,2009[72]		2008	2.5%(1.0%-5.9%)		
	XiaL,2010[77])		2009	1.2%(0.4%-3.7%)		
	Henan	1			0.5%(0.1%-3.2%)	
	(WuSX,2009[78])		2008	0.5%(0.1%-3.2%)		
	Hunan	1			6.8%(4.8%-9.4%)	
	(ChenLF,2009[83])		2009	6.8%(4.8%-9.4%)		
	Jiangsu	1			1.0%(0.2%-3.7%)	
	(WangYP,2009[87])		2007	1.0%(0.2%-3.7%)		

Our analysis reported high heterogeneities across the collected studies in evaluation of the pooled prevalence of the infections (HIV: *I*^2^ = 97.614, *p* < 0.001; HCV: *I*^2^ = 99.163, *p* < 0.001; HIV-HCV: *I*^2^ = 96.671; *p* < 0.001). For HIV and HCV infections, meta-regression demonstrated that these heterogeneities were mainly contributed by the geographical locations of the studies and contributions from study language, sample size, sampling method and study time period were not significant. In contrast, studies with sampling sizes greater than 500 tended to report higher HIV-HCV co-infection rates than otherwise and the influence of geographical location was marginal (Table 4). No publication bias was found across the 72, 71 and 19 studies reported HIV, HCV and HIV-HCV co-infection (Begg rank correlation analysis *p* = 0.163, 0.702 and 0.649, respectively). A significantly positive correlation (Spearman, *r* = 0.456, *p* < 0.001) was observed between HIV and HCV prevalence among clients in Chinese MMT during 2004–2010 countrywide.

**Table 4:** Result of individual variable meta-regression models for each stratified meta-analysis

	**Stratified meta-analyses**					
**Study Characteristic**	**HIV prevalence**		**HCV prevalence**		**HIV-HCV co-infection prevalence**	
	**Pooled estimate %**	**Meta-regression**	**Pooled estimate %**	**Meta-regression**	**Pooled estimate %**	**Meta-regression**
	**(95% CI), n**	**(β*, p*-value)**	**(95% CI), n**	**(β*, p*-value)**	**(95% CI), n**	**(β*, p*-value)**
Language of article:						
Chinese	6.0 (4.7-7.6), n = 70	0.060	60.1 (52.6-67.2), n = 69	0.018	4.2 (2.5-6.8), n = 18	0.402
English	8.4 (2.7-23.0), n = 2	*p* = 0.956	57.9 (35.9-77.1), n = 2	*p* = 0.964	13.5 (10.9-16.6), n = 1	*p* = 0.823
Sample size:						
< 500	4.8 (3.4-6.7), n = 50	0.178	59.8 (54.6-64.9), n = 53	−0.189	2.2 (1.2-4.1), n = 11	**1.367**
≥ 500	8.2 (5.6-11.8), n = 22	*p* = 0.561	60.3 (42.3-75.8), n = 18	*p* = 0.191	9.8 (5.7-16.2), n = 8	***p*** **= 0.029**
Study locations *:						
HTA	17.5 (14.0-21.6), n = 27	**−1.811**	70.2 (62.6-76.8), n = 21	**−0.335**	8.0 (4.8-12.9), n = 10	−0.831
LTA	2.4 (1.6-3.5), n = 45	***P*** **< 0.001**	55.5 (46.4-64.2), n = 50	***p*** **= 0.017**	2.5 (1.4-4.4), n = 9	*p* = 0.139
Sampling method:						
Cross-sectional	5.7 (4.3-7.4), n = 580	0.148	62.4 (54.2-69.9), n = 60	−0.275	4.6 (2.9-7.2), n = 19	-
Others	8.3 (5.1-13.2), n = 14	*p* = 0.686	46.2 (36.3-56.5), n = 11	*p* = 0.112	-	
Time period:						
2004–2006	8.0 (5.2-12.2), n = 16	−0.268	56.7 (47.0-65.8), n = 13	0.026	4.5 (0.3-40.4), n = 2	0.353
2007-2010	5.5 (4.1-7.3), n = 56	*p* = 0.458	60.9 (52.1-69.0), n = 58	*p* = 0.879	4.4 (2.7-7.2), n = 17	*p* = 0.803

Table showing the pool estimate (%), 95% confidence interval (CI), number of studies (n), meta-regression coefficient (β) and significance of β (*p*-value). *p*-values in bold print represent significant associations (*p* < 0.10).

* Study locations were categorized into two regions. HTA (high HIV transmission areas among DUs): Yunnan, Guizhou, Sichuan, Guangxi and Xinjiang; LTA (low HIV transmission areas among DUs): all provinces except above in Chinese mainland.

## Discussion

Consistent with findings from national sentinel surveillance [18], our meta-analyses indicated that HIV and HCV prevalence in MMT is distinctly higher in HTAs. This confirms that the prevalence of these infections remained highly concentrated among provinces along the traditional drug-trafficking routes but are considerably lower in the rest of the country.

Our estimated national HIV prevalence of 6.0% (95% CI: 4.7-7.7%) among people in MMT is not significantly different to the estimated HIV prevalence among non-MMT drug users (4.6-7.5%) reported by national sentinel surveillance during 2004–2009 [18]. We noticed that HIV prevalence among people in MMT in Sichuan (7.1%) and Guizhou (20.7%) province is higher than that from sentinel surveillance data [2,27,121-123], indicating that methadone clinics in these areas may have recruited more HIV-positive patients among drug users [124]. Additionally, our stratified time analysis of HIV, HCV and HIV-HCV co-infection among MMT clients did not show significant temporal trends in the prevalence of these infections since initiation of MMT in 2004. This suggested that the 2006 national policy to relax the eligibility criteria for MMT enrollment was not sufficient to encourage more HIV-infected drug users to participate in the program. MMT is known to significantly reduce the consumption of heroin and associated risk behaviours of the participants [24,125-129]. It is therefore in the best interest of the Chinese government to substantially scale-up the proportion of HIV-infected participants in MMT. The consequent reduction in risk behaviours of the infected population may help to confine the transmission sources of HIV infection. In order to achieve this, more comprehensive services, such as referral services, counseling, social supports targeting HIV-infected drug users etc., should be provided. Educational programs to reduce social stigma as well as psychological barriers for treatment adherence especially for HIV-infected drug users should be implemented.

Our analysis indicated a national HCV prevalence of 59.9% (95% CI: 52.7-66.7%) among MMT clients in China. This result is higher than the reported prevalence of 50.4% (95% CI; 42.5-58.4%) in a recent meta-analysis [130], in which the subjects were selected not only from MMT, but also from detoxification centers and the community. Chinese sentinel surveillance did not start monitoring HCV among drug users until 2009 and there have been no data currently published. Consistent with findings in other settings, HCV prevalence is positively correlated with HIV prevalence among MMT clients in China [89,130,131]. The fact that HCV prevalence was found to be 10 times higher than HIV prevalence may be due to a number of reasons. Biologically, transmission efficiency of blood-borne HCV has been estimated to be approximately 10 times greater than that of HIV for needle-stick injuries [132]. Secondly, HCV infection may have entered drug users population much earlier and hence already established a stabilized epidemic in China [131,133]. With a large number of chronic HCV-infected individuals in the drug user communities, a susceptible person may be more prone to the infection through needle-sharing with other IDUs.

The prevalence of HIV-HCV co-infection and HIV were not markedly different suggesting that a large proportion of HIV-infected MMT clients in China are also infected with HCV. Studies have shown that the co-infection of these diseases interact synergistically and the presence of each infection can substantially reduce the immune clearance of the other [134-136]. HCV co-infection may accelerate the progression to AIDS stage and death [134], even among people with continuous suppression of HIV replication [137]. It also blunts the CD4+ cell increase in HIV-infected people receiving antiretroviral therapy [137]. It is therefore a high priority to provide necessary healthcare and treatment for co-infected individuals in parallel to the roll-out of MMT in China.

Several limitations in this study should be noted. First, by the end of November 2010, there were 696 MMT clinics in China, covering 27 Chinese provinces [138], however, out of these, eight provinces did not publish any reports on the prevalence of HIV or HCV. Furthermore, estimates of HIV or HCV prevalence from Beijing, Henan, Hebei, Ningxia and Qinghai provinces were based on only one report. These may likely contribute to bias of the overall regional or national prevalence. Further investigations in more cities are necessary to provide a more accurate description of HIV and HCV epidemics. Second, our study was focused on HIV and HCV infections among the same study cohorts, studies reporting only HIV or HCV prevalence estimates were excluded in this review and hence some significant studies might be missing in the stratified meta-analyses. Third, there are also a potentially large number of governmental documents, community-level reports and other unpublished data that have never been archived in any of the public literature databases. Fourth, we identified that the presence of high heterogeneities may be attributable to demographical differences in HIV and HCV prevalence among MMT clients across different Chinese geographical regions, but not the language of article, sampling method and study time may not explain the variations observed. It is important to note that other unreported factors, such as age, socio-economic status and sexual behaviours, may also be contributable to the heterogeneities.

## Conclusions

Our study has several important implications to the future implementation of MMT in China. First, despite a rapid and substantial expansion of MMT program coverage in China [124,139] the proportion of HIV- and HCV-infected drug users attending MMT remains low. Although MMT is beneficial at an individual-level, it can potentially be an effective population-level intervention strategy. Future expansion of MMT should target the infected population, as only inclusion of a large proportion of these infected individuals may substantially reduce their risk behaviours and maximize the preventive effects of MMT at a population level. Second, MMT should not be viewed as a replacement for other harm reduction programs, such as, needle and syringe exchange programs (NSEPs). A previous study has demonstrated that NSEPs in China are both effective and cost-effective for HIV prevention [3]. MMT should be rolled-out in conjunction with NSEPs to reduce the number of new infections. Third, as a result of high percentage of HCV co-infection among HIV-infected MMT clients at baseline of the treatment, other programs that providing treatment to HIV and HCV infected patients should also be provided to MMT clients. Fourth, the greater risk of IDUs being infected with HIV and HCV and reported relapse to drug usage may be a contributing factor to low retention in MMT [140,141]. It is therefore important for MMT to ensure sufficient support and care is provided for enrolled IDUs to maintain their behavioural changes. Peer-group support and frequent counseling follow-ups may be beneficial in building motivating environment for abstinence from addictions.

## Competing interests

The authors declare that they have no competing interests.

## Author’s contributions

All authors were involved in the study design, including setting up the keywords search and project protocol. XZ and EPFC performed the literature search, quality assessment and data extraction. EPFC performed data analysis. YFW and YXL assisted with data collection, DPW and LZ assisted with data analysis and interpretation. ZX drafted the manuscript. LZ was responsible for the supervision of the project. All authors read and approved the final manuscript.

## Pre-publication history

The pre-publication history for this paper can be accessed here:

http://www.biomedcentral.com/1471-2334/12/130/prepub

## Supplementary Material

Additional file 1: **Figure S1.** Forest Plot showing the results of meta-analysis of HIV prevalence among clients in MMT (N = 72). **Figure S2.** Forest Plot showing the results of meta-analysis of HCV prevalence among clients in MMT (N = 71). **Figure S3.** Forest Plot showing the results of meta-analysis of HIV-HCV co-infection prevalence among clients in MMT (N = 19). **Table S1.** Quality assessment score of all studies [31,32,34-53,55-109,111,113-120]. (DOC 563 kb) Click here for file
