# Supplementary material for: HIV and HCV prevalence among entrants to methadone maintenance treatment clinics in China: a systematic review and meta-analysis
Source: BMC Infect Dis. 2012 Jun 8;12:130. doi: 10.1186/1471-2334-12-130 (PMC3434111; doi:10.1186/1471-2334-12-130)
Supplement: Additional file 1: — Figure S1. Forest Plot showing the results of meta-analysis of HIV prevalence among clients in MMT (N = 72). Figure S2. Forest Plot showing the results of meta-analysis of HCV prevalence among clients in MMT (N = 71). Figure S3. Forest Plot showing the results of meta-analysis of HIV-HCV co-infection prevalence among clients in MMT (N = 19). Table S1. Quality assessment score of all studies [31,32,34-53,55-109,111,113-120]. (DOC 563 kb) [file 1471-2334-12-130-S1.doc]

**SUPPLEMENTARY MATERIAL**

**Figure S1: Forest Plot showing the results of meta-analysis of HIV prevalence among clients in MMT (N=72).**

**Figure S2: Forest Plot showing the results of meta-analysis of HCV prevalence among clients in MMT (N=71).**

**Figure S3: Forest Plot showing the results of meta-analysis of HIV-HCV co-infection prevalence among clients in MMT (N=19).**

Table S1: Quality assessment score of all studies

|  | Q1 | Q2 | Q3 | Q4 | Q5 | Q6 | Q7 | Q8 | total |
| --- | --- | --- | --- | --- | --- | --- | --- | --- | --- |
| BaiY,2009 | 0 | 0 | 0 | 1 | 1 | 1 | 0 | 0 | 3 |
| CaiCP,2008 | 1 | 0 | 0 | 0 | 0 | 1 | 1 | 0 | 3 |
| ChangZR,2010 | 0 | 0 | 0 | 1 | 1 | 1 | 1 | 0 | 4 |
| ChenW,2009 | 1 | 0 | 1 | 1 | 1 | 0 | 1 | 0 | 5 |
| ChenA,2007 | 1 | 0 | 1 | 1 | 1 | 0 | 0 | 1 | 5 |
| ChenB,2009 | 0 | 0 | 0 | 1 | 1 | 0 | 0 | 0 | 2 |
| ChenC,2010 | 0 | 0 | 1 | 0 | 1 | 0 | 1 | 0 | 3 |
| ChenLF,2009 | 1 | 0 | 1 | 0 | 0 | 1 | 1 | 0 | 4 |
| DaiLP,2009 | 1 | 0 | 0 | 1 | 1 | 0 | 0 | 0 | 3 |
| DaiLP,2010 | 1 | 0 | 1 | 1 | 1 | 1 | 1 | 1 | 7 |
| DengCK,2009 | 1 | 0 | 1 | 1 | 1 | 0 | 0 | 1 | 5 |
| DongG,2009 | 0 | 0 | 0 | 1 | 1 | 1 | 0 | 0 | 3 |
| DuWJ,2007 | 1 | 0 | 0 | 1 | 1 | 1 | 1 | 0 | 5 |
| DuanYJ,2008 | 1 | 0 | 1 | 1 | 0 | 0 | 0 | 0 | 3 |
| DuJ,2009 | 1 | 1 | 0 | 0 | 1 | 0 | 1 | 0 | 4 |
| FangHR,2008 | 1 | 0 | 0 | 1 | 1 | 0 | 0 | 0 | 3 |
| FengSQ,2010 | 1 | 0 | 1 | 1 | 1 | 0 | 0 | 0 | 4 |
| FengYH,2010 | 1 | 1 | 0 | 1 | 1 | 0 | 0 | 0 | 4 |
| FuLP,2007 | 1 | 0 | 0 | 1 | 1 | 0 | 0 | 0 | 3 |
| FuYF,2009 | 1 | 1 | 0 | 1 | 1 | 1 | 0 | 0 | 5 |
| GaoLF,2010 | 1 | 0 | 0 | 1 | 1 | 0 | 1 | 0 | 4 |
| HanXJ,2010 | 1 | 1 | 0 | 1 | 0 | 0 | 1 | 0 | 4 |
| HaoC,2006 | 1 | 0 | 1 | 1 | 1 | 0 | 0 | 0 | 4 |
| HaoC,2009 | 1 | 0 | 1 | 1 | 1 | 0 | 0 | 0 | 4 |
| HaoXQ,2009 | 1 | 0 | 0 | 1 | 1 | 1 | 0 | 0 | 4 |
| HeHX,2008 | 1 | 0 | 0 | 0 | 1 | 1 | 1 | 0 | 4 |
| HeXX,2010 | 1 | 1 | 0 | 0 | 1 | 0 | 1 | 0 | 4 |
| HuWS,2010 | 1 | 0 | 1 | 1 | 1 | 0 | 0 | 0 | 4 |
| JiangA,2009 | 1 | 1 | 0 | 1 | 1 | 0 | 0 | 0 | 4 |
| JiaW,2008 | 1 | 0 | 1 | 1 | 1 | 0 | 0 | 1 | 5 |
| LiLY,2009 | 1 | 1 | 0 | 1 | 1 | 0 | 0 | 0 | 4 |
| LiT,2010 | 0 | 1 | 0 | 0 | 1 | 1 | 1 | 1 | 5 |
| LiuXY,2009 | 0 | 1 | 0 | 1 | 1 | 0 | 0 | 0 | 3 |
| LiuHB,2010 | 1 | 0 | 0 | 1 | 1 | 1 | 1 | 0 | 5 |
| LiuJB,2006 | 1 | 0 | 1 | 1 | 1 | 1 | 0 | 0 | 5 |
| LiuJK,2009 | 1 | 0 | 0 | 1 | 1 | 0 | 0 | 0 | 3 |
| LiuXP,2010 | 1 | 0 | 0 | 1 | 1 | 1 | 1 | 0 | 5 |
| LiuY,2009 | 1 | 0 | 0 | 1 | 1 | 0 | 0 | 0 | 3 |
| LiXL,2008 | 1 | 0 | 1 | 1 | 1 | 0 | 0 | 0 | 4 |
| LiXL,2009 | 1 | 0 | 0 | 1 | 1 | 0 | 0 | 1 | 4 |
| LiXY,2009 | 1 | 0 | 0 | 1 | 1 | 0 | 0 | 0 | 3 |
| LiYC,2009 | 1 | 0 | 1 | 1 | 1 | 1 | 0 | 0 | 5 |
| LiYF,2009 | 1 | 0 | 1 | 0 | 1 | 1 | 1 | 0 | 5 |
| PengJS,2007 | 1 | 0 | 1 | 0 | 1 | 0 | 0 | 0 | 3 |
| QianHZ,2007 | 1 | 0 | 1 | 1 | 1 | 1 | 0 | 1 | 6 |
| QiuXQ,2009 | 0 | 0 | 0 | 1 | 1 | 0 | 0 | 0 | 2 |
| ReZW,2009 | 1 | 0 | 1 | 1 | 0 | 0 | 0 | 0 | 3 |
| ShenL,2009 | 1 | 0 | 1 | 1 | 1 | 0 | 0 | 0 | 4 |
| SongHB,2010 | 1 | 0 | 0 | 1 | 1 | 1 | 0 | 0 | 4 |
| SuMF,2010 | 1 | 0 | 0 | 1 | 1 | 1 | 1 | 0 | 5 |
| TangXY,2007 | 1 | 1 | 1 | 1 | 1 | 1 | 0 | 0 | 6 |
| TanXL,2007 | 0 | 0 | 0 | 1 | 1 | 0 | 0 | 0 | 2 |
| WangY,2009 | 1 | 0 | 0 | 1 | 1 | 0 | 1 | 0 | 4 |
| WangCQ,2009 | 1 | 0 | 1 | 1 | 1 | 0 | 0 | 0 | 4 |
| WangDY,2009 | 1 | 0 | 0 | 1 | 1 | 1 | 0 | 0 | 4 |
| WangDY,2010 | 1 | 1 | 0 | 0 | 1 | 0 | 0 | 1 | 4 |
| WangJ,2008 | 1 | 0 | 1 | 1 | 0 | 0 | 0 | 1 | 4 |
| WangJ,2010 | 1 | 0 | 0 | 1 | 1 | 0 | 0 | 1 | 4 |
| WangM,2009 | 0 | 1 | 0 | 1 | 0 | 1 | 0 | 0 | 3 |
| WangWM,2010 | 0 | 1 | 0 | 0 | 0 | 1 | 1 | 0 | 3 |
| WangXR,2007 | 1 | 1 | 0 | 0 | 0 | 1 | 0 | 0 | 3 |
| WangYP,2009 | 1 | 1 | 0 | 0 | 1 | 1 | 0 | 0 | 4 |
| WuGH,2010 | 1 | 0 | 0 | 1 | 1 | 1 | 1 | 0 | 5 |
| WuZL,2010 | 1 | 0 | 0 | 1 | 1 | 0 | 0 | 0 | 3 |
| WuLH,2007 | 1 | 1 | 1 | 0 | 1 | 1 | 0 | 1 | 6 |
| WuSX,2009 | 1 | 0 | 1 | 1 | 0 | 0 | 1 | 0 | 4 |
| XiaL,2010 | 1 | 1 | 0 | 1 | 0 | 1 | 0 | 1 | 5 |
| XiaX,2010 | 1 | 0 | 1 | 1 | 1 | 0 | 0 | 0 | 4 |
| XuGY,2008 | 1 | 0 | 0 | 1 | 0 | 0 | 0 | 0 | 2 |
| XueHM,2010 | 0 | 0 | 0 | 1 | 1 | 0 | 0 | 0 | 2 |
| XuYP,2007 | 1 | 1 | 1 | 0 | 0 | 1 | 0 | 1 | 5 |
| YangGW,2010 | 1 | 0 | 1 | 1 | 1 | 0 | 0 | 0 | 4 |
| YangYC,2011 | 1 | 0 | 0 | 1 | 1 | 1 | 1 | 0 | 5 |
| YaoW,2008 | 1 | 0 | 1 | 1 | 0 | 0 | 0 | 0 | 3 |
| YuanL,2010 | 1 | 0 | 1 | 1 | 1 | 0 | 0 | 0 | 4 |
| YuanZX,2010 | 1 | 0 | 1 | 1 | 1 | 1 | 1 | 0 | 6 |
| ZangJF,2010 | 1 | 0 | 0 | 1 | 1 | 0 | 1 | 1 | 5 |
| ZhangHF,2009 | 1 | 0 | 0 | 1 | 1 | 0 | 0 | 1 | 4 |
| ZhangMH,2010 | 1 | 0 | 0 | 1 | 1 | 0 | 1 | 1 | 5 |
| ZhangMJ,2008 | 1 | 0 | 0 | 1 | 1 | 0 | 0 | 1 | 4 |
| ZhangQL,2008 | 1 | 0 | 0 | 1 | 1 | 0 | 1 | 1 | 5 |
| ZhangXH,2008 | 0 | 0 | 0 | 1 | 1 | 0 | 0 | 0 | 2 |
| ZhangXH,2007 | 1 | 0 | 1 | 1 | 1 | 0 | 0 | 0 | 4 |
| ZhanSW,2008 | 1 | 0 | 1 | 1 | 1 | 0 | 0 | 0 | 4 |
| ZhaoXH,2009 | 1 | 0 | 1 | 1 | 1 | 0 | 0 | 0 | 4 |
| ZhengWX,2009 | 1 | 0 | 0 | 1 | 1 | 0 | 1 | 0 | 4 |
| ZhouX,2009 | 1 | 0 | 0 | 1 | 1 | 0 | 0 | 0 | 3 |
| ZhouXP,2009 | 1 | 0 | 0 | 0 | 1 | 1 | 1 | 1 | 5 |
| ZhuangHY,2008 | 1 | 0 | 0 | 1 | 0 | 1 | 0 | 0 | 3 |
| ZhuXH,2010 | 1 | 0 | 1 | 1 | 1 | 1 | 1 | 0 | 6 |
